# Early Detection and Quality Indicators: Assessing the Clinical Impact and Effectiveness of Lowering the Colorectal Cancer Screening Age

**DOI:** 10.7759/cureus.78911

**Published:** 2025-02-12

**Authors:** Mena Louis, Adeel Akhtar, Bolaji Ayinde, Nathaniel Grabill, Edward Foxhall, Emily Murdoch, Daniel Sarmiento Garzon

**Affiliations:** 1 General Surgery, Northeast Georgia Medical Center Gainesville, Gainesville, USA; 2 Internal Medicine, Northeast Georgia Medical Center Gainesville, Gainesville, USA; 3 Surgery, Northeast Georgia Medical Center Gainesville, Gainesville, USA; 4 Trauma and Acute Care Surgery, Northeast Georgia Medical Center Gainesville, Gainesville, USA; 5 Colorectal Surgery, Northeast Georgia Medical Center Braselton, Braselton, USA

**Keywords:** adenoma detection rate, colonoscopy outcomes, colorectal cancer screening, epidemiology of crc, lowered screening age, preventive healthcare, public health, quality indicators, screening guidelines

## Abstract

Objective

With colorectal cancer (CRC) being a leading cause of cancer-related deaths globally, early detection through screening is critical for improving survival rates. Recent guidelines recommend lowering the initial screening age from 50 to 45 years based on increasing CRC incidence among younger adults.

Methodology

This retrospective study evaluates the impact of this adjustment on adenoma detection rates (ADR), a validated quality indicator of screening colonoscopies. We conducted a retrospective analysis of 2,792 patients who underwent colonoscopy screening at a major healthcare institution. Patients were categorized into three age groups: 45-49, 50-59, and 60-75 years. We compared ADR across these groups with a focus on evaluating the consistency of ADR in the newly included younger age group against established benchmarks.

Result

The ADRs for the 45-49 age group were 51.2% (105/205) in male patients and 38.5% (116/301) in female patients, closely mirroring those of the 50-59 age group at 51.08% (188/368) in male patients and 34.80% (133/382) in female patients, with no significant reduction in detection rates. Gender-specific analysis revealed higher ADR in male patients across all age groups. These findings were statistically significant when comparing procedure type with age group and gender (p < 0.05).

Conclusion

The study supports maintaining the already established ADR for the new age group of 45-49 as the ADR was comparable to those in the older groups. Similar polyp biopsy +/- removal rates in the 45-49-year-old age group (43.6%; 221/506) compared to the older age group (42.7%; 320/750) is also an indirect measure of the effectiveness of early screening for colon cancer.

## Introduction

Colorectal cancer (CRC) remains a significant public health challenge globally, ranking as the third most common cancer and the second leading cause of cancer-related deaths [1,2]. Early detection through screening colonoscopies significantly improves prognosis by identifying precancerous lesions, namely adenomas, which, if untreated, may progress to malignant tumors [3,4]. Recent shifts in epidemiological trends have shown an alarming rise in CRC incidence among younger adults, prompting a reevaluation of screening guidelines [5]. While the exact cause of this increase remains unclear, several factors have been proposed. Changes in diet, particularly increased consumption of processed foods and red meat, along with rising obesity rates and sedentary lifestyles, have been linked to a higher risk of early-onset CRC. Additionally, alterations in gut microbiota and increasing exposure to environmental carcinogens may contribute to this trend. Some studies also suggest that younger adults may experience delayed diagnosis due to lower suspicion of CRC in this population, leading to more advanced disease at presentation. In response, many health authorities, including the U.S. Preventive Services Task Force, have revised their recommendations to lower the initial screening age from 50 to 45 years [6]. This pivotal adjustment aims to intercept the disease earlier, potentially improving outcomes for a broader demographic [7].

The effectiveness of colonoscopy screenings is often gauged by adenoma detection rates (ADR), a critical quality indicator validated by numerous surgical and medical societies [8]. ADR reflects the percentage of screened individuals with at least one adenomatous polyp identified, with current benchmarks set at 20% for females and 30% for males [8,9]. These rates are not mere statistical measures; they are benchmarks that signify the thoroughness of mucosal inspection and indirectly correlate with the potential to reduce CRC incidence through proactive intervention [10].

Despite the broad acceptance of ADR as a cornerstone of quality in colonoscopy screenings, introducing a younger screening cohort poses new questions: Does the existing threshold for ADR apply equally well to this younger age group? Are this group's biological and epidemiological characteristics sufficient to warrant reassessing what constitutes an 'adequate' ADR? This study delves into these questions by comparing ADR across different age groups, focusing on the newly recommended age group (45-49 years) against the established older groups. By examining ADR within these cohorts, the research seeks to validate the quality of colonoscopy performed and assess the appropriateness of current ADR benchmarks in reflecting the nuances of an evolving demographic landscape [11].

This inquiry is crucial not just for its clinical implications but also for its potential to influence public health policy. As screening guidelines shift to include younger populations, understanding the dynamics of ADR across age groups will help ensure that the measures of quality and effectiveness remain robust, safeguarding the goals of early detection and prevention in CRC management. Our comprehensive analysis aims to provide evidence-based insights that could drive future policy decisions and clinical practices, aligning them more closely with the shifting epidemiology of this serious disease.

## Materials and methods

Study design and setting

This retrospective cohort study was conducted at Northeast Georgia Health System, a large healthcare institution in Gainesville, GA, with a well-established CRC screening program. The study aimed to evaluate the impact of lowering the recommended age for CRC screening from 50 to 45 years on ADR. By comparing ADR between the newly recommended age group (45-49 years) and the previously established groups (50-59 years and 60-75 years), the study sought to determine whether current ADR benchmarks are appropriate for this younger population.

Population and data collection

The study included 2,792 patients who underwent colonoscopy screening at the institution between January 2019 and December 2021. Patients were stratified into three age groups: 45-49, 50-59, and 60-75 years. Data were extracted from electronic health records (EHRs), including demographic details (age, sex, ethnicity, and race), colonoscopy findings, and procedure reports.

Inclusion criteria consisted of patients aged 45 years or older at the time of their first screening colonoscopy during the study period, with complete colonoscopy findings, including data on polyp detection and removal. Patients were excluded if they had a history of CRC, inflammatory bowel disease, or prior colon surgery to minimize confounding factors affecting ADR calculations.

Colonoscopy procedure

All colonoscopies were performed by board-certified gastroenterologists or general and colorectal surgeons with extensive experience in colorectal screening. The quality of bowel preparation was evaluated using the Boston Bowel Preparation Scale (BBPS), and only procedures with a BBPS score of 2 or higher (on a scale of 0 to 3) were included to ensure optimal visualization. Procedures were categorized into three types based on outcomes: colonoscopy with biopsy (at least one polyp biopsied but not removed), polyp removal via snare technique (a polyp detected and removed during the procedure), and screening-only colonoscopy (no polyp detected or removed).

Outcome measures

The primary outcome measure was adenoma detection rate (ADR), defined as the proportion of screening colonoscopies resulting in the detection, biopsy, or removal of at least one adenomatous polyp. Since individual pathology reports were not accessible within the dataset used for this analysis, ADR was indirectly estimated based on the assumption that two-thirds of biopsied polyps are adenomatous. Secondary analyses evaluated differences in procedure types based on gender and race, including the distribution of procedures involving biopsy, polyp removal, and screening-only colonoscopies.

Statistical analysis

All statistical analyses were conducted using MedCalc statistical software (www.mdcalc.com). Descriptive statistics summarized patient demographics, including age, gender, race, and ethnicity. Chi-squared tests were employed to compare categorical variables across age groups, gender, and race, with a p-value of less than 0.05 considered statistically significant. Additionally, chi-squared tests for trends were performed to assess any significant trends in ADR across age groups, and logistic regression was used to adjust for potential confounders such as sex and race. This robust approach ensured an accurate evaluation of the relationships between age, gender, and ADR.

Ethical considerations

The study protocol was reviewed and approved by the Institutional Review Board (IRB) at the healthcare institution. Given its retrospective design, the IRB granted a waiver of consent. All data were de-identified prior to analysis to ensure compliance with the Health Insurance Portability and Accountability Act (HIPAA) and to protect patient privacy and confidentiality.

## Results

Data from 2,792 patients who underwent screening colonoscopies between January 2019 and December 2021 were analyzed. The distribution of patients by age group and gender is summarized in (Table 1). Patients were stratified into three age groups: 506 (18.1%) were in the 45-49 age group, 750 (26.9%) in the 50-59 age group, and 1,536 (55.0%) in the 60-75 age group.

**Table 1:** Patient demographics and clinical characteristics (N = 2,792) ADR: adenoma detection rates

Demographics	n	Percentage (%)
Age group (years)		
45-49	506	18.10%
50-59	750	26.90%
60-75	1,536	55.00%
Gender		
Male	1,373	49.20%
Female	1,419	50.80%
Race		
White	2,201	79.00%
Black	362	13.00%
Asian	57	2.00%
Other	167	6.00%

**Figure 1:**
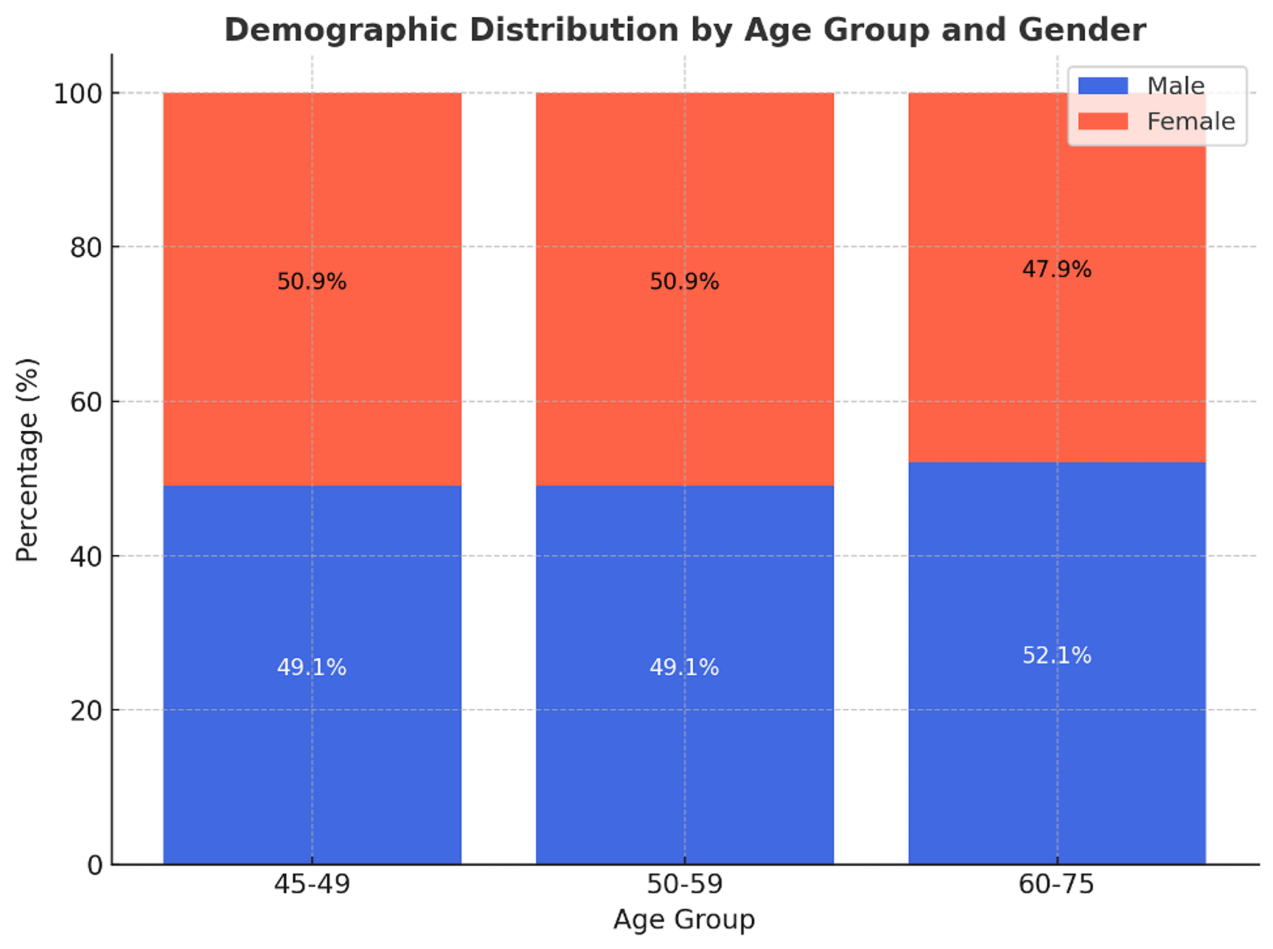
Demographic distribution by age group and gender

The gender distribution within the 50-59 age group was relatively balanced, with 368 male patients (49.1%) and 382 female patients (50.9%). In the 60-75 age group, there was a slightly higher proportion of male patients (n=800; 52.1%) compared to female patients (n=736; 47.9%) (Figure 1).

The racial distribution of the study population showed that 2,201 (79.0%) were White, 362 (13.0%) were Black, 57 (2.0%) were Asian, and 167 (6.0%) were classified as other races.

**Figure 2:**
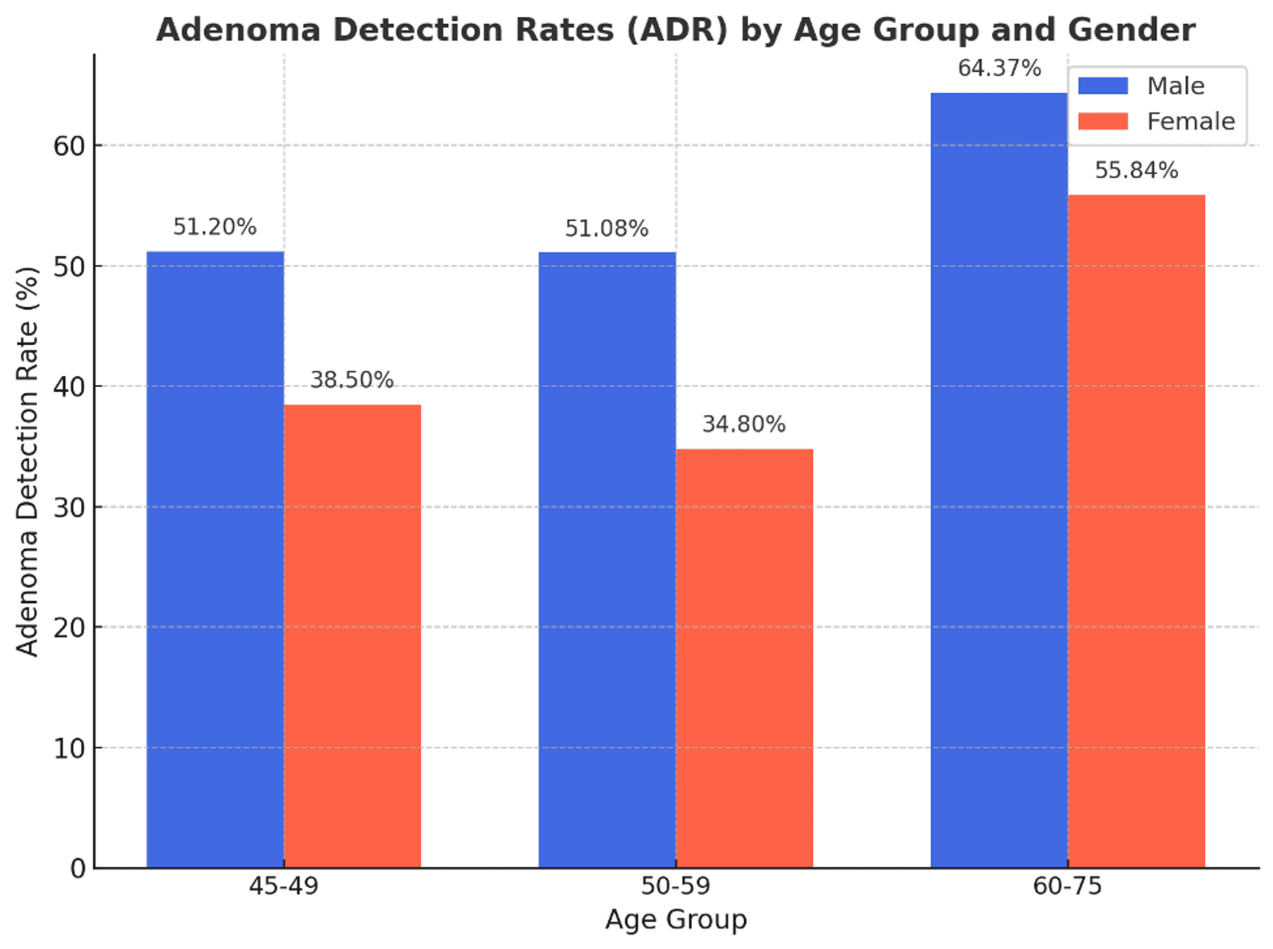
ADR by age group and gender ADR: adenoma detection rates

The ADRs were analyzed by age group and gender (Table 2). In the 45-49 age group, 51.2% of male patients (105/205) and 38.5% of female patients (116/301) had at least one polyp detected and either biopsied or removed (Figure 2). The chi-squared test for this age group revealed a p-value of 0.0183, indicating statistically significant gender differences in ADR.

**Table 2:** ADR by age group and gender ADR: adenoma detection rate; CI: confidence interval

Age group (years)	Gender	Total screenings (n)	Colonoscopy with biopsy (n)	ADR (%)	95% CI
45-49	Male	273	157	31.0%	27.5 - 34.7%
	Female	233	146	26.5%	22.8 - 30.3%
50-59	Male	399	199	26.5%	23.3 - 29.9%
	Female	351	198	26.4%	22.9 - 30.3%
60-75	Male	701	624	40.6%	37.4 - 43.9%
	Female	835	610	39.7%	36.5 - 43.0%

In the 50-59 age group, 51.08% of male patients (188/368) and 34.80% of female patients (133/382) showed positive ADRs. The chi-squared test for this age group returned a p-value of less than 0.0001, further highlighting significant gender disparities in adenoma detection.

In the 60-75 age group, 64.37% of male patients (515/800) and 55.84% of female patients (411/736) had polyps detected. The chi-squared test for this demographic showed a p-value of 0.0006, affirming significant differences between male and female patients with ADRs (Table 2).

Chi-squared tests for trends

**Figure 3:**
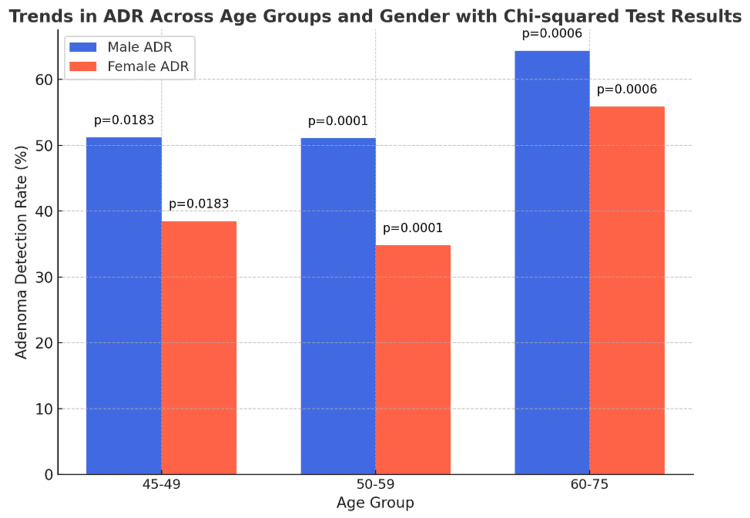
Trends in ADR across age groups and gender, along with the chi-squared test results. ADR: adenoma detection rates

Further analysis using chi-squared tests for trends across age groups provided additional insights. In the 45-49 year age group, the chi-squared test for trend yielded a p-value of 0.0183, indicating a statistically significant variation in polyp detection rates across genders (Figure 3).

In the 50-59 age group, a pronounced trend was observed with a p-value of 0.0001, indicating substantial disparities in adenoma detection trends between male and female patients.

Finally, in the 60-75 age group, the test for trend resulted in a p-value of 0.0006, confirming a notable trend in polyp detection rates across genders in this older cohort.

Comparative analysis of ADR across age groups

**Figure 4:**
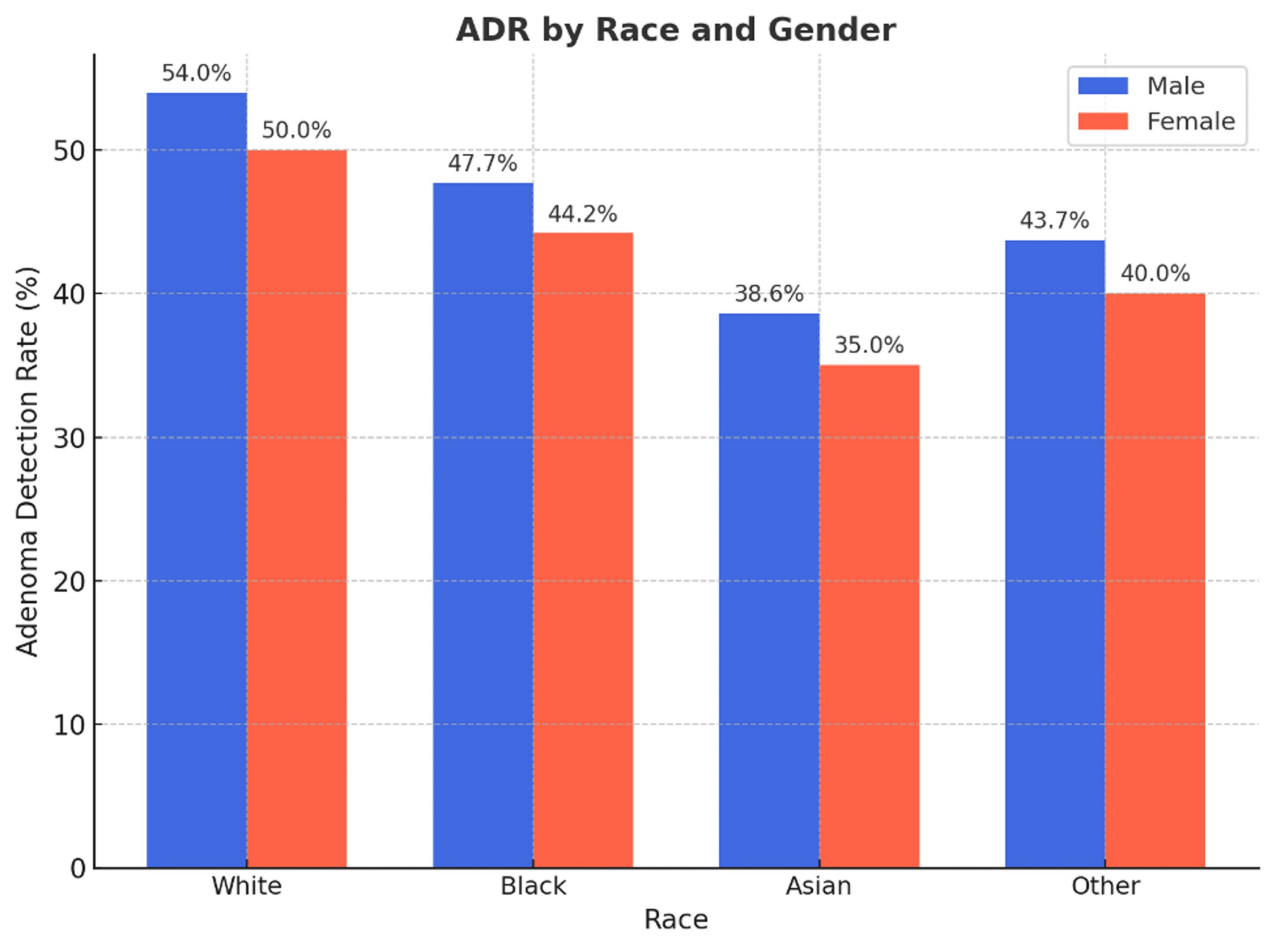
ADR by race and gender, displaying the adenoma detection rates for male and female patients across racial groups (White, Black, Asian, Other). ADR: adenoma detection rates

The ADRs in the 45-49 age group were notably similar to those in the 50-59 age group, suggesting that the quality of colonoscopy, as measured by ADR, remains consistent across these age ranges. Despite introducing a younger screening cohort, the established ADR benchmarks of 20% for female patients and 30% for male patients were shown to be appropriate and effective for assessing the quality of colonoscopy screenings in the younger age group (Figure 4).

## Discussion

This study demonstrates that lowering the colorectal cancer (CRC) screening age to 45 years is an effective strategy that does not compromise screening quality. Adenoma detection rates (ADR) in the 45-49 age group were comparable to those in the 50-59 age group, reinforcing the appropriateness of extending screening eligibility to younger adults. Detecting precancerous lesions in younger populations provides a critical opportunity to intervene earlier, potentially reducing CRC incidence and mortality [1]. Given the increasing incidence of CRC in individuals under 50, early screening may help mitigate the trend of late-stage diagnoses often seen in younger patients [2,3].

ADR remains an essential quality metric for colonoscopy and has been extensively validated across different age groups [3]. The results of this study confirm its reliability even in the 45-49 age group. However, ADR benchmarks were originally developed based on older populations, and their applicability to younger patients remains a subject of discussion. The slightly lower ADR observed in female patients aged 45-49, compared to the benchmark, suggests the need for further investigation into whether current standards adequately reflect demographic variations in risk [8]. Future research should determine if adjustments in ADR benchmarks for younger patients, particularly female patients, would improve screening effectiveness.

Consistent with previous research, this study found significant gender differences in ADR, with male patients exhibiting higher rates across all age groups [9,10]. Male patients have been shown to develop adenomas at a younger age and in greater numbers, which may be attributed to differences in hormonal influence, dietary factors, or genetic predisposition [11]. These findings reinforce the need for gender-specific approaches in CRC screening. While male patients may require more frequent or earlier screening, female patients may benefit from additional risk stratification methods to optimize screening recommendations. Studies exploring sex-based biological mechanisms driving these differences may enhance personalized screening strategies.

The results of this study strongly support lowering the CRC screening age to 45. Expanding eligibility allows for earlier detection of adenomas, which has long been associated with a decreased risk of CRC progression. This aligns with recent guideline changes by the U.S. Preventive Services Task Force and other major organizations [8]. However, despite these recommendations, uptake of CRC screening among younger adults remains suboptimal due to a combination of provider hesitancy, lack of patient awareness, and insurance barriers [6]. Addressing these challenges through public health initiatives and policy adjustments could improve early detection efforts.

Additionally, racial and ethnic disparities in CRC incidence and outcomes remain a pressing concern. Studies have shown that Black individuals are more likely to develop CRC at an earlier age and present with more advanced disease at diagnosis. However, our study did not find higher ADR in Black patients compared to White patients, contrary to prior research suggesting a greater prevalence of advanced adenomas in this population. These findings indicate the need for further investigation into potential barriers to screening and variations in adenoma prevalence across racial groups.

This study has several limitations. The retrospective design limits the ability to establish causality between screening age and ADR. The lack of pathology data necessitated indirect estimation of ADR using the assumption that two-thirds of biopsied polyps are adenomatous, which may introduce variability. Having direct access to biopsy results would provide a more accurate assessment. Additionally, since the study was conducted at a single institution, the findings may not be fully generalizable to broader populations. Future research involving multicenter data and diverse patient cohorts will help validate these results.

Further studies should assess the long-term impact of screening at age 45 versus 50, particularly in reducing CRC incidence and mortality. Investigating the biological and environmental factors contributing to rising CRC rates in younger populations is essential. Additionally, refining screening guidelines based on demographic variations--such as gender and race--could lead to more precise screening recommendations. The role of non-invasive screening methods, including stool-based tests and emerging molecular markers, should also be explored as complementary strategies to improve early detection and accessibility.

By demonstrating that ADR in the 45-49 age group is comparable to older cohorts, this study reinforces the effectiveness of early screening while emphasizing the need for ongoing evaluation of quality indicators. Ensuring that screening guidelines remain responsive to epidemiological trends and demographic differences will be essential in reducing CRC burden and improving patient outcomes.

## Conclusions

This study provides compelling evidence that lowering CRC screening age to 45 years maintains the quality of screening, with ADRs in the 45-49 age group mirroring those of the 50-59 age group. The findings support recent guideline adjustments and highlight the potential of earlier screening to enhance CRC prevention. Gender-specific differences in ADR emphasize the need for refined, gender-tailored screening practices. Our study reinforces ADR as a reliable quality indicator for colonoscopies and suggests that benchmarks may need adjustment for different demographic segments. As CRC incidence rises among younger adults, these guideline changes are crucial for reducing incidence and mortality and adapting healthcare systems to ensure high-quality screening for all. This study validates the effectiveness of early screening initiation and calls for continued research and public health strategies to prioritize early detection and intervention.

## References

[1] Dekker E, Tanis PJ, Vleugels JL, Kasi PM, Wallace MB (2019). Colorectal cancer. Lancet.

[2] Baidoun F, Elshiwy K, Elkeraie Y (2021). Colorectal cancer epidemiology: Recent trends and impact on outcomes. Curr Drug Targets.

[3] Gupta S (2022). Screening for Colorectal Cancer. Hematol Oncol Clin North Am.

[4] Bretthauer M, Løberg M, Wieszczy P (2022). Effect of colonoscopy screening on risks of colorectal cancer and related death. N Engl J Med.

[5] Burnett-Hartman AN, Lee JK, Demb J, Gupta S (2021). An update on the epidemiology, molecular characterization, diagnosis, and screening strategies for early-onset colorectal cancer. Gastroenterology.

[6] Shaukat A, Levin TR (2022). Current and future colorectal cancer screening strategies. Nat Rev Gastroenterol Hepatol.

[7] Ladabaum U, Dominitz JA, Kahi C, Schoen RE (2020). Strategies for colorectal cancer screening. Gastroenterology.

[8] Corley DA, Jensen CD, Marks AR (2014). Adenoma detection rate and risk of colorectal cancer and death. N Engl J Med.

[9] Spadaccini M, Schilirò A, Sharma P, Repici A, Hassan C, Voza A (2023). Adenoma detection rate in colonoscopy: how can it be improved?. Expert Rev Gastroenterol Hepatol.

[10] He X, Lv X, Zhang B, Ying X, Hu C, Zhou X, Hu J (2023). Adenoma detection rate in average-risk population: an observational consecutive retrospective study. Cancer Control.

[11] Kim I, Lee HH, Ko YJ (2022). Factors associated with the risk of colorectal neoplasia in young adults under age 40. Korean J Intern Med.

